# Ubiquitin-mediated regulation of autophagy

**DOI:** 10.1186/s12929-019-0569-y

**Published:** 2019-10-21

**Authors:** Ruey-Hwa Chen, Yu-Hsuan Chen, Tzu-Yu Huang

**Affiliations:** 1 Academia Sinica, Institute of Biological Chemistry, Taipei, 115 Taiwan; 2 Institute of Biochemical Sciences, College of Life Science, National Taiwan University, Taipei, 100 Taiwan

**Keywords:** Protein ubiquitination, Autophagy, Selective autophagy, Ubiquitin E3 ligase, Deubiquitinating enzyme

## Abstract

Autophagy is a major degradation pathway that utilizes lysosome hydrolases to degrade cellular constituents and is often induced under cellular stress conditions to restore cell homeostasis. Another prime degradation pathway in the cells is ubiquitin-proteasome system (UPS), in which proteins tagged by certain types of polyubiquitin chains are selectively recognized and removed by proteasome. Although the two degradation pathways are operated independently with different sets of players, recent studies have revealed reciprocal cross talks between UPS and autophagy at multiple layers. In this review, we summarize the roles of protein ubiquitination and deubiquitination in controlling the initiation, execution, and termination of bulk autophagy as well as the role of ubiquitination in signaling certain types of selective autophagy. We also highlight how dysregulation of ubiquitin-mediated autophagy pathways is associated with a number of human diseases and the potential of targeting these pathways for disease intervention.

## Introduction

Ubiquitin-proteasome system (UPS) and autophagy are two major cellular degradation machineries in eukaryotes, both of which are crucial in eliminating misfolded/unfolded proteins to maintain cell and tissue homeostasis and to prevent aging-related changes and a plethora of human diseases. In general, short-lived and soluble misfolded/unfolded proteins are targeted by UPS, whereas long-lived and insoluble protein aggregates are eliminated by autophagy [1, 2]. The cargos of autophagy are not limited to proteins and include dysfunctional or superfluous organelles. Although the two systems are operated independently, recent studies have revealed multiple layers of interconnections between UPS and autophagy. For instance, inhibition of UPS leads to a compensatory stimulation of autophagy via several mechanisms, whereas autophagy inhibition activates or impairs proteasomal flux depending on the cellular and environmental conditions [3, 4]. In addition, components of either system can serve as the proteolytic targets of the other system [4]. In this review, we chose to focus on the role of protein ubiquitination in regulating autophagy. Other aspects of the crosstalk between UPS and autophagy have been reviewed elsewhere [4, 5].

### Overview of protein ubiquitination

Ubiquitination is a posttranslational modification involving the conjugation of the 76 amino acid ubiquitin to the lysine residue of other proteins. This modification is mediated by the sequential action of E1 ubiquitin-activating enzyme, E2 ubiquitin conjugating enzyme, and E3 ubiquitin ligase [6]. The removal of ubiquitin from the substrate is catalyzed by a class of deubiquitinating enzymes (DUBs) [7]. Ubiquitin contains seven lysine residues and one N-terminal methionine residue, each of which can be attached to another ubiquitin moiety. As a consequence, proteins can be modified by ubiquitin monomer or polymer with different length and linkage types, making ubiquitination as one of the most elaborate and versatile posttranslational modifications [8–10]. In the homotypic polyubiquitination, all building blocks of the chain are connected through the same lysine or methionine residue and a total of eight different chain types can be formed. To add the complexity, heterotypic chain, which contains more than one linkage types, can also be formed and can be further categorized into mixed and branched chains. Importantly, these structurally distinct ubiquitin modifications are recognized by effector proteins with linkage-specific ubiquitin-binding domains to result in diverse functional outcomes, such as degradation, signal transduction, and alteration in subcellular localization. For instance, K48- and K11-linked chains are pivotal signals for proteasomal degradation, whereas K6, K27, K33, K63, and linear chains are usually of a nondegradative fate [8–10]. Recent studies have further revealed the role of branched ubiquitin chain in changing the nondegradative to degradative fate or in the enhancement of degradative signal [10–13].

### Overview of autophagy

Autophagy is a lysosome-based degradation program activated by various cellular stresses including nutrient/energy starvation, hypoxia, ER stress, hypoxia, and organelle damage. During autophagic process, double-membrane vesicles, termed autophagosomes, are formed in the cytoplasm to sequester cellular components. This is followed by fusion of autophagosome with lysosome and degradation/recycling of sequestered cellular components to generate macromolecular building blocks [2, 14, 15]. The prime functions of autophagy include the removal of harmful substances (such as damaged organelles, protein aggregates, and intracellular pathogens), adaptation to metabolic stresses, and renovation during differentiation and development. Dysfunction of autophagic process has been associated with numerous diseases, including infectious diseases, cancer, neurodegeneration, cardiovascular disorders, and aging [16–18].

In the past decade, the molecular mechanisms of autophagy have been intensively studied. Autophagy initiation is governed by the ULK1 serine/threonine kinase which forms a complex with FIP200, ATG13, and ATG101 [19, 20]. Upon various cellular stresses, ULK1 is activated, resulting in the phosphorylation of multiple downstream factors to trigger the autophagy cascade. One effector of ULK1 is the class III PI3K complex, which contains the lipid kinase VPS34 and regulator proteins Beclin-1, VPS15, and ATG14 [21]. ULK1 promotes the activation and the recruitment of class III PI3K complex to the autophagosome formation site (phagophore), where it generates PI3P to function in autophagosome nucleation [22]. ATG9, the only transmembrane protein in the core autophagic machinery, is thought to supply membrane to autophagosome [23]. Additionally, ATG9 binds ATG2 and WIPI proteins (ATG18 in yeast, the PI3P effectors), to participate in the early stage of autophagosome biogenesis from ER [24]. Further expansion and completion of the autophagosome depends on the two ubiquitin-like conjugation systems [25]. The ATG12 conjugation system is responsible for the conjugation of ubiquitin-like protein ATG12 to ATG5, which in turn forms a complex with ATG16L1. The ATG12-ATG5-ATG16L1 complex functions as the E3 ligase for the second conjugation system, in which the ubiquitin-like LC3 subfamily proteins (ATG8 in yeast) are conjugated to the membrane-residing phosphatidylethanolamine (PE). PE modification of the LC3 family proteins are essential for the elongation and closure of autophagosome membrane. To achieve autophagic degradation, autophagosome needs to fuse with lysosome or late endosome. The fusion requires UVRAG-containing class III PI3K complex (also known as the PI3K complex II), tethering factors such as HOPS complex, SNARE proteins such as STX17 on autophagosome membrane and VAMP8 and SNAP29 on endosome/lysosome, RAB proteins such as RAB7, and the LC3 family proteins [26]. After fusion, the inner membrane of autophagosome and materials wrapped in the inner membrane are degraded and the resulting small molecules are recycled to the cytosol.

### Regulation of autophagy induction by ubiquitination and deubiquitination

Induction of autophagy needs to be tightly controlled for cells to cope with various stressed conditions. Reversible ubiquitination of the core autophagy induction factors, i.e., the subunits of ULK1 and PI3K complexes, has been revealed as a common mechanism for turning on and off the autophagy process under different cellular contexts. In addition, ubiquitination participates in positive feedback regulations for timely induction of autophagy.

#### The role of E3 ligases

**Fig. 1:**
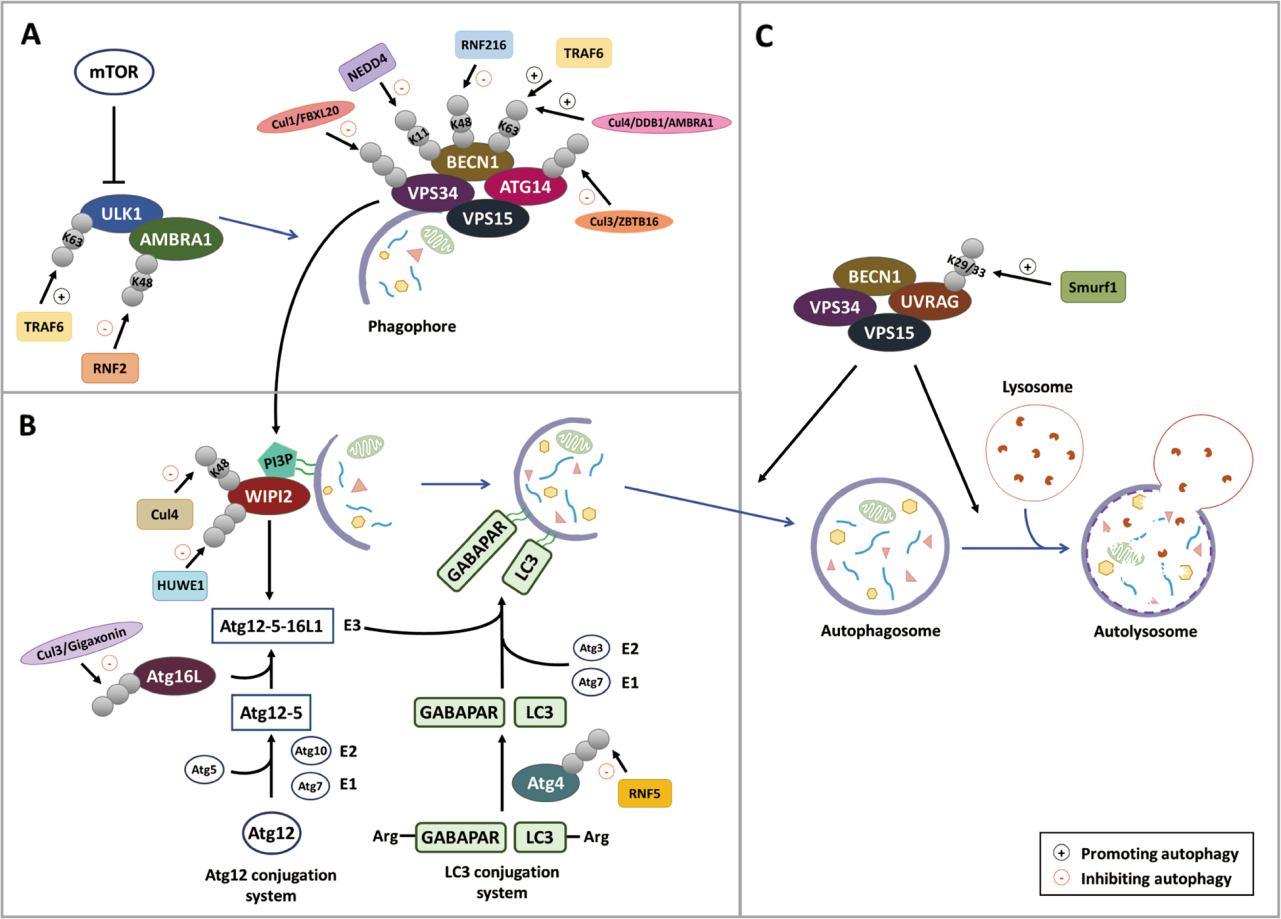
Functional roles of ubiquitin ligases in regulating autophagy. Summary of the proteins acting in the initiation (**a**), autophagosome biogenesis (**b**), and autophagosome maturation (**c**) steps of the autophagic process that are subjected to ubiquitination by various E3 ligases. The ubiquitin chain types and the effect of ubiquitination on autophagy (promotion or inhibition) are indicated

The ubiquitin ligase TRAF6, which mediates the formation of K63-linked ubiquitin chain, plays important roles in autophagy induction. TRAF6 promotes K63 ubiquitination of ULK1, thereby enhancing ULK1 stability and function [27]. Notably, recruitment of ULK1 to TRAF6 requires the cofactor AMBRA1, a subunit of class III PI3K. Since ULK1 phosphorylates and activates AMBRA1, the TRAF6-mediated ULK1 ubiquitination participates in a positive feedback mechanism to potentiate autophagy initiation. TRAF6 also catalyzes the K63 ubiquitination of Beclin-1 [28]. This ubiquitination occurs at the BH3 domain of Beclin-1 and thus blocks Beclin-1 interaction with Bcl-2 to promote autophagy in response to the activation of Toll-like receptor (TLR) 4. Under starvation, Beclin-1 K63 ubiquitination is mediated by Cul4 E3 ligase with AMBRA1 as a substrate adaptor, thereby promoting autophagy [29]. However, the role of AMBRA1 in regulating autophagy initiation is complex. Another study reported that AMBRA1 is transiently dissociated from Cul4 at an early stage of autophagy induction. The released AMBRA1 inhibits Cul5 ubiquitin ligase, thereby stabilizing mTOR inhibitor DEPTOR [30]. Since ULK1 activity is required for the dissociation of AMBRA1 from Cul4, this regulation of AMBRA1 binding partner establishes a feedback mechanism for a rapid autophagy induction. In contrast to TRAF6 and AMBRA1, the ubiquitin ligases NEDD4 and RNF216 promote Beclin-1 proteasomal degradation to inhibit autophagy by assembling K11- and K48-linked ubiquitin chains on Beclin-1, respectively [31, 32]. Other autophagy inducing factors also undergo degradable ubiquitination. For instance, AMBRA1 K48 ubiquitination is promoted by RNF2 E3 ligase, leading to its proteasomal degradation [33]. VPS34 and ATG14 ubiquitination and degradation are mediated by Cul1 E3 ligase containing FBXL20 and Cul3 E3 ligase containing ZBTB16 in response to DNA damage and G protein coupled signaling, respectively [34, 35]. Together, subunits of the ULK1 and VPS34 complexes are targeted by multiple ubiquitin ligases (Fig. 1a). While K63 ubiquitination promotes autophagy induction in response to stressed conditions or accelerates autophagy initiation through feedback mechanisms, ubiquitination by K48- and K11-linked chain types impairs autophagy induction through degradation of the core autophagic proteins.

#### The roles of DUBs

**Fig. 2:**
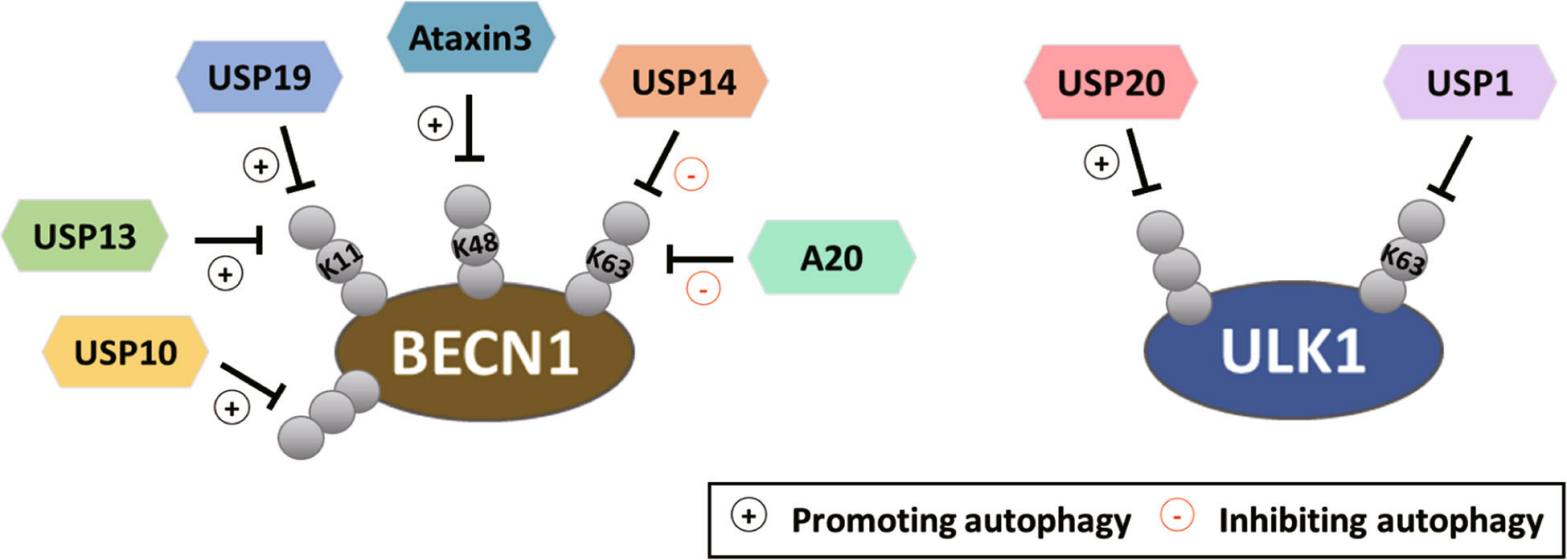
Functional roles of DUBs in regulating autophagy initiation. Summary of DUBs that regulate autophagy initiation by targeting ULK1 or Beclin-1. The ubiquitin chain types and the effect of ubiquitination on autophagy (promotion or inhibition) are indicated

Among the autophagy inducing factors, Beclin-1 is a popular target for ubiquitination. Similarly, Beclin-1 appears as a hub for DUB-mediated regulation (Fig. 2). A20, which specifically targets the K63-linked ubiquitin chain, antagonizes the function of TRAF6 on modifying Beclin-1, thereby attenuating autophagy induction in response to TLR signaling [28]. Belcin-1 K63 ubiquitination is also negatively controlled by USP14, resulting in autophagy inhibition. Importantly, USP14 is itself activated by Akt-mediated phosphorylation and this mechanism contributes to the inhibition of autophagy activity by Akt [36]. Several DUBs influence on Beclin-1 degradable ubiquitination. For instance, USP10 and USP13 reduce Beclin-1 ubiquitination to prevent its degradation. The function of USP10 and USP13 is reversed by a chemical compound, spautin-1, which inhibits autophagy by promoting Beclin-1 degradation. Interestingly, Beclin-1 positively controls the stability of USP10 and USP13, suggesting the existence of a feedback mechanism to maintain Beclin-1 level [37]. Beclin-1 stabilization is also promoted by USP19 and ataxin 3, which specifically removes K11- and K48-ubiquitin chain from Belcin-1, respectively [38, 39]. Finally, Beclin-1 is indirectly regulated by DUB USP33, which deubiquitinates Beclin-1 partner RALB [40]. This deubiquitination event is important for the binding of RALB with the exocyst component EXO84 and Beclin-1, which in turn drives the assembly of active ULK1 and Beclin-1-VPS34 complex for autophagy initiation [41]. The ability of Belcin-1 to be targeted by multiple DUBs highlights the importance of reversible ubiquitination in regulating autophagy initiation under different cellular contexts, even though the upstream signals regulating these deubiquitination events and the specific ubiquitin ligases that counteract these DUBs remain mostly uncharacterized.

Besides Beclin-1, ULK1 is regulated by DUBs (Fig. 2). ULK1 K63 ubiquitination is antagonized by USP1. This function of USP1, however, regulates ULK1 cellular compartmentalization by promoting ULK1 localization to the Triton X-100 soluble fraction. Depletion of USP1 or inhibition of USP1 activity by small molecular inhibitor leads to the formation of ULK1 insoluble aggregates which also contain p62 and the aggregation marker HDAC6, thereby inhibiting canonical autophagic flux but promoting lysosome-mediated degradation of p62 [42]. The degradable ULK1 ubiquitination is reversed by USP20. Under basal conditions, USP20 maintains ULK1 level to facilitate autophagy initiation. Under prolonged starvation, the binding of USP20 to ULK1 is diminished, leading to autophagy inhibition [43]. Thus, ULK1 deubiquitination could control the dynamics of autophagy process and the decision between canonical and nonconventional autophagy.

### Regulation of autophagosome biogenesis by ubiquitination

A key event for autophagosome biogenesis is the recruitment of PI3P-binding proteins to phagophore, such as WIPI2. This is followed by phagophore recruitment of ATG12-ATG5-ATG16L1 complex for the lipidation of LC3 family proteins and subsequent autophagosome expansion [44, 45]. Recent studies have revealed that both WIPI2 and ATG16L1 are subjected to ubiquitin-mediated regulation (Fig. 1b). Ubiquitination of ATG16L1 is mediated by gigaxonin [46], a substrate adaptor of Cul3 ubiquitin ligase mutated in a neurodegenerative disease called giant axonal neuropathy [47]. Interestingly, gigaxonin-mediated ubiquitination promotes ATG16L1 degradation through both proteasomal and autophagic routes and ATG16L1 aggregates are accumulated in gigaxonin knockout neurons. As to WIPI2, the ubiquitin ligase HUWE1 is responsible for its ubiquitination and proteasomal degradation. Importantly, targeting WIPI2 to HUWE1 requires mTORC1-dependent phosphorylation on S395 of WIPI2, uncovering a link of mTORC1 to WIPI2 degradation for autophagy inhibition [48]. During mitosis, WIPI2 ubiquitination and degradation are also potentiated. This is mediated by the Cul4 family of ubiquitin ligase, whose activity is elevated in mitosis due to increased Cul4 neddylation. Importantly, the reduction of autophagy activity in mitosis through WIPI2 ubiquitination is important for the proper progression of mitotic phase, as restoration of WIPI2 during mitosis induces mitotic slippage and cell senescence [49]. Thus, WIPI2 ubiquitination is regulated by nutrient availability and cell cycle to influence on autophagy activity.

The LC3 family protein GABARAP is itself a ubiquitin-like protein. Interestingly, GABARAP can also be modified by K48-linked ubiquitin chain through the activity of centrosome-residing ubiquitin ligase Mib1 [50] (Fig. 1 b). The centriolar satellite protein PCM1, however, binds GABARAP to protect it from Mib1-mediated ubiquitination and degradation. This stabilization of GABARAP allows it trafficking along with PCM1 from centrosome reservoir to phagophore during starvation, thereby facilitating the formation of GABARAP-positive autophagosome. Thus, the centriolar satellite controls GABARAP ubiquitination and trafficking to regulate autophagosome biogenesis.

The cysteine protease ATG4 is responsible for processing the LC3 to facilitate its lipidation and for deconjugating LC3-II at the final step of autophagy [51, 52]. The membrane-associated ubiquitin ligase RNF5 targets a specific membrane pool of ATG4B for ubiquitination and degradation (Fig. 1 b), thereby limiting LC3 processing to restrict autophagy activity in basal condition. Upon starvation or alteration in cell redox states, the binding of RNF5 to ATG4B is attenuated, which contributes to autophagy induction [53].

### The emerging role of ubiquitination in autophagosome maturation

The fusion of autophagosome with lysosome is required for autophagic flux. EPG5, a RAB7A effector, is localized to late-endosome/lysosome and promotes their fusion with autophagosome by binding to LC3 [54]. USP8, a DUB localized to the endocytic compartment, binds EPG5 and removes K63-linked ubiquitin chain from EPG5 [55]. This deubiquitination event enhances EPG5 binding to LC3, thus potentiating autophagic flux to maintain the identity of embryonic stem cell. The E3 ligase responsible for EPG5 K63 ubiquitination remains undetermined. UVRAG, a subunit of class III PI3K complex specifically required for autophagosome maturation, is modified by K29/K33 non-canonical ubiquitin chain by E3 ligase SMURF1 [56] (Fig. 1c). This ubiquitination decreases the binding of UVRAG with its inhibitor Rubicon, thereby enhancing autophagic flux. Hence, these ubiquitination events on EPG5 and UVRAG mainly affect their interaction with other proteins, rather than promoting degradation.

### Ubiquitin controls autophagy termination

**Fig. 3:**
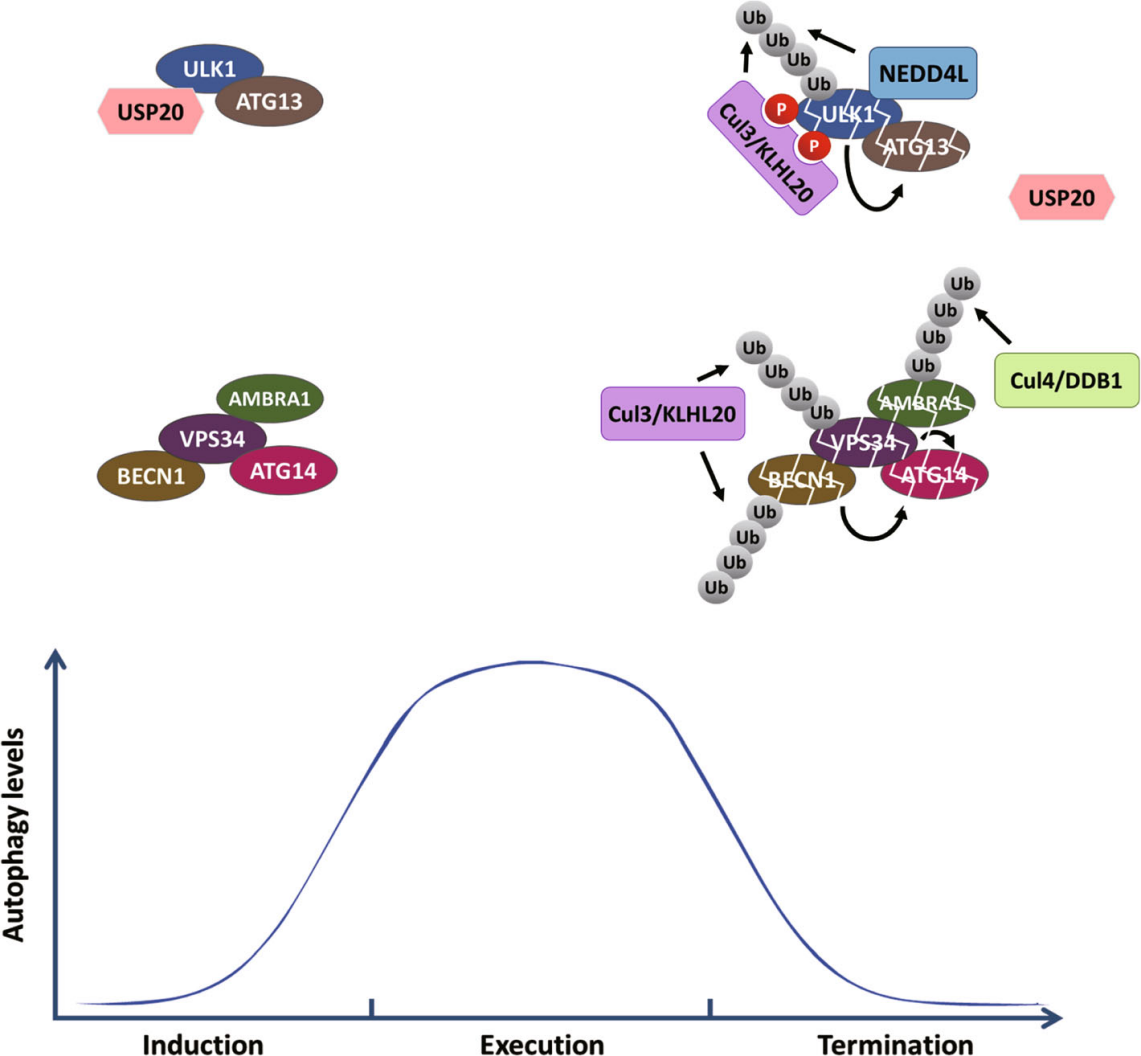
Mechanisms for ubiquitin-mediated autophagy termination. The ULK1 and VPS34 complexes are stable in the induction phase of autophagy. After the execution phase of autophagy, several components of the two complexes are degraded via direct or indirect action of indicated E3 ligases, thereby contributing to autophagy termination

Autophagy is a self-limiting process. It is turned on for cell to cope with various stressed conditions. However, once the stressed situation is resolved, autophagy machinery needs to be turned off to avoid excessive degradation. Ubiquitin-mediated degradation appears to be an ideal mechanism for terminating a cellular process, such as autophagy. Indeed, several ubiquitin-based mechanisms are involved in time-dependent or feedback regulation for autophagy termination (Fig. 3).

The WD40 protein AMBRA1 acts as a component for both class III PI3K complex and Cul4 ubiquitin ligase complex. AMBRA1 undergoes Cul4-dependent self-ubiquitination and degradation. However, at the early stage of autophagy induction, AMBRA1 is transiently dissociated from Cul4, rendering its stabilization. AMBRA1 re-associates with Cul4 at later time points to result in its downregulation. This mechanism contributes in part to autophagy termination as expression of a Cul4-binding deficient AMBRA1 mutant leads to a prolonged autophagy response [30].

Besides Cul4 ubiquitin ligase, the Cul3 ubiquitin ligase containing KLHL20 as the substrate adaptor is found to play a major role in autophagy termination. Upon autophagy induction, the Cul3-KLHL20 complex specifically targets the autophosphorylated ULK1 for ubiquitination and degradation. Additionally, KLHL20 is recruited to phagophore where it binds and ubiquitinates VPS34 and Beclin-1. Furthermore, other subunits of the ULK1 and VPS34 complexes, such as ATG13 and ATG14, are also degraded after KLHL20-mediated degradation of their partners, even though they are not direct substrates of KLHL20. Thus, KLHL20 participates in feedback regulations for promoting the degradation of multiple autophagy inducing factors after the induction of autophagy. Depletion of KLHL20 or expression of an autophosphorylation-defective ULK1 mutant leads to a prolonged autophagy response and an increased cell death under starvation [57].

The HECT family ubiquitin ligase NEDD4L and DUB USP20 also participate in autophagy termination. During prolonged starvation, NEDD4L catalyzes the K27 and K29 ubiquitination on ULK1 [58], whereas the interaction between USP20 and ULK1 is attenuated [43]. Both mechanisms lead to downregulation of ULK1 protein level. Thus, multiple E3 ligases and DUB act in concert to limit ULK1 protein abundance, thereby contributing to autophagy termination. Importantly, the *ULK1* mRNA is consistently present and its translation is induced when mTOR is reactivated by the release of building blocks from the autolysosome. This mechanism allows the recovery of ULK1 protein level for the next run of autophagy induction [58].

### Ubiquitin signaling in selective autophagy

#### Overview of selective autophagy

**Fig. 4:**
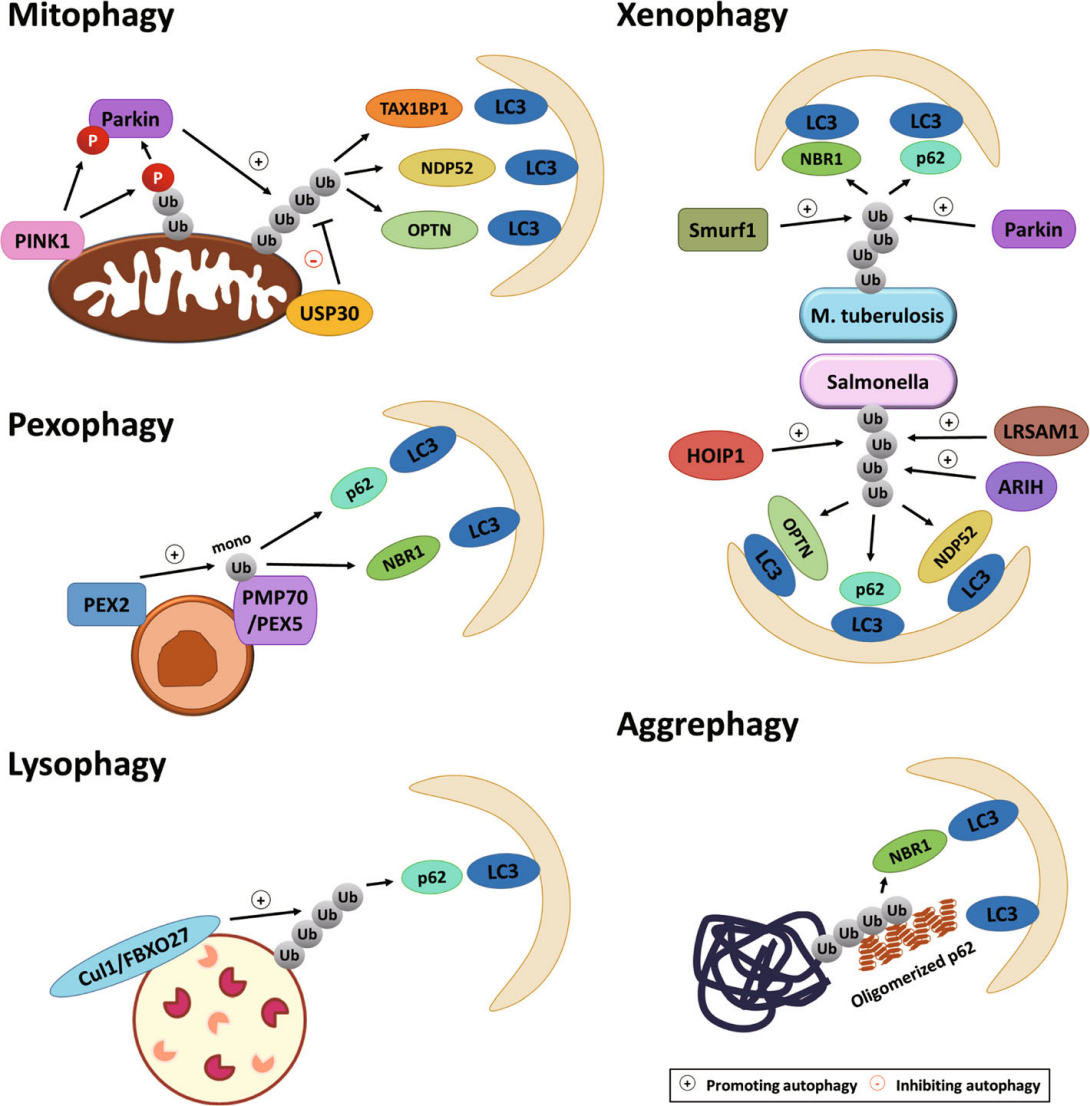
Ubiquitin-dependent selective autophagy. Summary of the molecular mechanisms of major types of selective autophagy using protein ubiquitination as a mark of the cargo. The E3 ligases and DUB involved in generating or removing the ubiquitin chain and the autophagy adaptors used to link ubiquitinated cargos to LC3 are indicated

Autophagy was originally considered as a nonselective bulk degradation process, but numerous studies have later reported the selective degradation of various cellular organelles or substances via autophagy mechanism, including mitochondria, ER, peroxisome, lipid droplet, ribosome, midbody, nucleus, protein aggregate, and specific pathogens [59]. In theory, selective autophagy should result in a more specific removal of damaged or harmful cellular components and thus could be more important in disease prevention than bulk autophagy. To achieve selectivity, the cargos are often linked to LC3 family proteins directly or indirectly via ubiquitin-dependent or independent mechanisms. This review focuses only on the ubiquitin-dependent selective autophagy. Different from the bulk autophagy where protein ubiquitination often plays a modulating role, protein ubiquitination in many types of selective autophagy serves as a mark for cargo recognition and a signal for process initiation. Ubiquitinated proteins generated on the surface of cargos are responsible for the recruitment of specific autophagy adaptor proteins (also known as autophagy receptors), such as p62, OPTN, NBR1, NDP52, and TAX1BP1 [60, 61]. Since these autophagy adaptors possess both ubiquitin-binding domain and LC3-interacting region (LIR), they function as bridges to recruit LC3 to the cargos. Certain autophagy adaptor, such as NDP52, also recruits upstream autophagy initiating complex to the cargos [62, 63]. In this way, autophagy machinery generates autophagosome to specifically engulf the cargos. Below, we discuss the role of ubiquitination in the initiation and regulation of several types of selective autophagy (Fig. 4).

#### Mitophagy

The best studied ubiquitin-dependent selective autophagy mechanism is mitophagy, in which the protein kinase PINK1 and E3 ligase Parkin play a key role in building the ubiquitin chains on the outer surface of damaged mitochondria. Upon mitochondria damage, PINK1 is stabilized on mitochondria membrane to recruit Parkin [64–66] and phosphorylates the S65 residue on both ubiquitin and the UBL domain of Parkin, which act in concert to activate Parkin on mitochondria [67–69]. Parkin in turn catalyzes the ubiquitination of numerous mitochondrial outer membrane proteins [70, 71]. Recent studies indicate that these ubiquitinated proteins not only facilitate the recruitment of autophagy adaptors but also serve as PINK1 substrates to establish a feedforward mechanism for reinforcing the PINK1-Parkin pathway [68, 72]. Quantitative proteomic study identified numerous mitochondrial proteins whose ubiquitination is dependent on Parkin [73]. Furthermore, multiple ubiquitin chain types, such as K6, K11, K48 and K63 are generated following mitochondrial depolarization [68]. It is generally believed that the identity of the substrates is less important than the density of ubiquitin chains on mitochondria to determine the onset of mitophagy [74]. Consequently, autophagy adaptors are recruited to the damaged mitochondria. CRISPR-mediated knockout analysis on HeLa cells revealed that OPTN, NDP52 and TAX1BP1 are redundantly required for mitophagy, with OPTN playing the most prominent role [75]. OPTN further recruits TBK1 to promote mitophagy through a feedback mechanism [76, 77]. Nevertheless, other study indicated the crucial role of p62 in Parkin-dependent autophagy in mouse macrophages and embryonic fibroblasts [78, 79]. It is unclear whether this discrepancy is owing to the difference in the relative abundance of these adaptors in different cell types.

Besides Parkin, mitophagy can be regulated by other factors that influence on the ubiquitination of mitochondrial membrane proteins. USP30, a transmembrane DUB localized on the mitochondrial outer membrane, antagonizes the function of Parkin by removal of ubiquitin chains from mitochondria [80]. Interestingly, USP30 undergoes a Parkin-dependent monoubiquitination and proteasomal degradation, thus establishing a feedforward mechanism for Parkin to promote mitophagy. Additionally, E3 ligases other than Parkin that target mitochondrial fusion and fission machineries [81, 82] can also regulate mitophagy, as damaged mitochondria need to go through a fission process to be enclosed into the autophagosome [83].

#### Pexophagy

Peroxisomes are ubiquitous organelles involving in modulation of metabolic responses and redox regulation [84]. In mammals, damaged peroxisomes are removed through ubiquitin-dependent selective autophagy pathway [85]. Consistently, an increase in ubiquitinated proteins on the surface of peroxisomes induces pexophagy. Peroxisome membrane proteins PEX5 and PMP70 are targeted for monoubiquitination under stressed conditions through the peroxisome E3 ligase PEX2 [86]. As to the autophagy adaptors, p62 and NBR1 act in a cooperated fashion to link ubiquitinated peroxisome to autophagic machinery [85, 87].

#### Lysophagy

Although bulk autophagy and selective autophagy require the fusion with lysosome for autophagic flux, damaged lysosome is itself removed by an autophagic process called lysophagy. Lysophagy utilizes a ubiquitin-dependent selective autophagy mechanism, as ubiquitinated proteins, p62, and LC3 are all found on the surface of damaged lysosomes [88, 89]. The damaged lysosome membranes are also decorated with galectin-3 [89], which is presumably due to the exposure of the luminal proteins to the cytosol side following membrane rupture. Recent study indicates that FBXO27, a membrane localized substrate adaptor of Cul1 ubiquitin ligase, catalyzes the ubiquitination of N-glycoproteins exposed to the damaged lysosome, thereby facilitating the recruitment of autophagy adaptor p62 [90].

#### Xenophagy

In addition to cellular organelles, ubiquitin-dependent selective autophagy is also exploited to eliminate intracellular pathogens such as *Salmonella, Listeria,* and *Mycobacterium*, a process called xenophagy [91]. In the host cells, these pathogens are quickly marked by ubiquitin chains on their surface. Multiple host E3 ligases are reported to ubiquitinate pathogens. For instance, Smurf1 and Parkin are involved in the ubiquitination of *M. tuberculosis* [92, 93]. LRSAM1, ARIH, and HOIPI complex are responsible for *Salmonella* ubiquitination [23, 94, 95]. Of note, the ubiquitin chain types generated by these E3 ligases are different. While LRSAM1 generates K6 and K27 chains, ARIH and HOIP1 form K48 chain and M1 chain, respectively. These different ubiquitin chains are clustered to form distinct foci on bacteria surface [96]. The M1 chain specifically recruits OPTN, whereas the recruitment of p62 and NDP52 to bacteria is independent of M1 chain, demonstrating their non-redundant functions [97]. In addition to inducing xenophagy, the M1 chain on bacteria activates NF-kB pathway to promote proinflammatory cytokine secretion, thereby inhibiting bacteria proliferation [96, 97].

#### Aggrephagy

Aggrephagy is induced in response to various proteotoxic conditions, such as inhibition of proteasome or chaperons and interference with productive translation, in which aggregates of ubiquitinated proteins are observed [98]. Formation of such aggregates requires p62 [99]. Recent studies indicate that p62 drives the aggregate formation via a process called liquid-liquid phase separation [61, 100]. In addition to the ubiquitin binding domain (UBA), p62 contains a oligomerization domain (PB1). Oligomerization of p62 allows a high-avidity binding of ubiquitinated proteins via UBA domain and finally condenses the ubiquitinated proteins into larger structures. Subsequently, P62 tethers LC3 to the condensates through its LIR to facilitate a selective sequestration of ubiquitin condensates to the autophagosome. Other autophagy adaptor, such as NBR1, can also contribute to the condensation by interacting with p62 [101].

Since ubiquitinated proteins can also be targeted to undergo proteasomal degradation, one intriguing question is how to distinguish the autophagy fate from proteasome fate. Although p62-mediated condensation may be a determining factor to direct ubiquitinated proteins to the autophagy pathway, it is worth noting that p62 can also function as a direct adaptor to recruit ubiquitinated proteins to the proteasome in cytosol or nucleus [102, 103]. Another possibility for determining the fate of ubiquitinated protein is the quality of ubiquitin chains. It is thought that K48-ubiquitinated proteins are degraded by proteasome, whereas K63 chain modified proteins are substrates of aggrephagy. However, M1, K63, and K48 chains can all trigger phase separation in vitro via binding to p62, albeit with a lower efficiency than the K48 chain [61, 100]. Perhaps the nature of aggrephagy substrates do not have much difference from those of the proteasome substrates and, rather, the high concentration of ubiquitin chains determines the aggrephagy fate by favoring a p62-mediated phase separation [101].

### Ubiquitin-mediated autophagy regulation in diseases

#### Neurodegenerative diseases

There is increasing evidence for the association of autophagy dysfunction with various neurodegenerative diseases, such as Alzheimer’s disease, tauopathies, Parkinson’s disease (PD), polyglutamine disorders, and amyotrophic lateral sclerosis [104]. The most well-known neurodegenerative disease associated with defects in ubiquitin-mediated autophagy is PD, which is the second most common late-onset neurodegenerative disease resulted from the loss of dopaminergic neurons in the substantia nigra pars compacta. Mutations in genes encoding either PINK1 or Parkin are associated with autosomal recessive forms of PD [105]. Mice deficient in either Parkin or PINK1 exhibit mitochondrial impairments, but most of them cannot recapitulate the prime features of human PD, that is, loss of dopaminergic neurons [106, 107]. A recent study generated by Parkin homozygous knockout in the background of mice with the expression of a proof-reading defective mtDNA polymerase (called mutator mice). The combination of Parkin knockout and mtDNA mutation leads to the loss of dopaminergic neurons selectively in the substantia nigra and motor defect [108]. This genetic evidence, in conjunction with the mitochondrial dysfunction found in brain and other organs of PD patients [106], point out the importance of mitophagy in PD etiology.

Another type of neurodegenerative disorder is caused by mutant proteins with the expansion of continuous stretches of glutamine (called polyQ repeats), including Huntington’s disease, spinocerebellar ataxia (SCA), and spinal and bulbar muscular atrophy [104]. A recent study uncovered a link of ubiquitin-mediated autophagy regulation to various polyQ diseases. Ataxin 3 is a polyQ-containing DUB and its polyQ expansion is associated with SCA type 3, in which neurodegeneration occurs in the striatum and cerebellum [109]. Interestingly, the normal function of ataxin 3 is to remove the polyubiquitin chain from Beclin-1, leading to its stabilization [38]. With this function, ataxin 3 is required for starvation-induced autophagy. Importantly, several proteins with expanded polyQ repeats, including ataxin 3 itself, can compete with ataxin 3 for binding Beclin-1, in a polyQ length-dependent fashion. Furthermore, although ataxin 3 with expanded polyQ repeats elicits higher binding affinity to Beclin-1, it is defective in removing ubiquitin chain from Beclin-1. Thus, these findings identify a link of ataxin 3 to autophagy regulation and, more importantly, suggest that impairment of Beclin-1-mediated autophagy accounts for one mechanism of polyQ repeat-associated neurodegenerative diseases.

#### Infectious diseases and inflammation

As described above, ubiquitin serves as a tag to facilitate the autophagic degradation of intracellular pathogens (xenophagy) and a number of ubiquitin E3 ligases are involved in the addition of such tag. Since autophagy core machinery is also required for the xenophagy process, regulators that affect ubiquitin-dependent turnover of autophagic core factors could also control xenophagy. For instance, RNF216, which targets Beclin-1 for ubiquitination and degradation, promotes *Listeria monocytogenes* proliferation and distribution in cell and mouse models [32]. Nevertheless, it should be noted that the bulk autophagy could elicit housekeeping function to restrict inflammation, thereby favoring pathogen survival [91]. The balance between selective autophagy and anti-inflammation could determine the outcome of infection and immunological functions. One example for ubiquitination-mediated balance of anti-infection arm and anti-inflammation arm lies in USP19-depedent Beclin-1 deubiquitination [39]. On one hand, this deubiquitination stabilizes Beclin-1 to favor autophagy-dependent pathogen clearance. On the other hand, the stabilized Beclin-1 binds to the CARD domain of MAVS to prevent MAVS-RIG-I association, thereby inhibiting type I interferon production and anti-viral immunity.

#### Liver disease, metabolic syndromes and cancer

Autophagy is important in controlling hepatocyte lipid metabolism to maintain normal liver functions [110]. Autophagy deficiency by ATG7 knockout aggravates liver steatosis induced by high fat diet and promotes the development of liver adenoma [111]. Conversely, liver steatosis impairs autophagy through ATG7 downregulation [112]. One important function of autophagy to regulate lipid metabolism is the turnover of lipid droplets via a selective autophagy process called lipophagy [111]. Similar to other selective autophagy processes, lipophagy requires certain core autophagic factors. A recent study reveals an inhibitory role of HUWE1-mediated WIPI2 degradation in lipid droplet turnover in the liver, leading to the accumulation of liver neural lipids [48]. Besides liver disease, ubiquitin-mediated autophagy regulation is implicated in other metabolic syndromes. For instance, failure of autophagy termination by KLHL20 deficiency potentiates muscle atrophy in diabetes mouse model [57].

Autophagy plays complex roles in cancer, which may depend on the different stages of cancer development. In the tumor initiating stage, autophagy suppresses carcinogenesis. However, once tumor is formed, tumor cells exploit the autophagic process for them to survive in the harsh environments [17]. The impact of ubiquitin-mediated autophagy regulation on tumor formation and progression is poorly studied. A recent study reported that the Smurf1-induced UVRAG ubiquitination promotes not only autophagosome maturation but hepatocellular carcinoma (HCC) growth [56]. Furthermore, phosphorylation of UVRAG at S522, which disrupts Smurf1 binding, correlates with poor survival of HCC patients. These findings support a tumor suppressive role of autophagy in HCC.

## Conclusion and perspectives

In this review, we discussed the impact of protein ubiquitination in autophagy regulation. Protein ubiquitination serves as an ‘eat me’ signal for many types of selective autophagy by recruiting autophagic adaptors and subsequently the core autophagic proteins. In contrast to the “signaling” role of ubiquitination in selective autophagy, protein ubiquitination mainly plays modulating role in almost every step of bulk autophagy. The initiation and nucleation steps of autophagosome formation are most prevalently regulated by ubiquitination, meaning that ubiquitination controls the onset of autophagic process in response to various stressed conditions. Nevertheless, later steps of autophagosome formation and autophagosome maturation are also subjected to ubiquitin-mediated regulation. Furthermore, ubiquitin-mediated protein turnover has been used as a prime mechanism for autophagy termination under prolonged stress conditions, thereby preventing the detrimental effect of excessive autophagic degradation. The pleiotropic role of protein ubiquitination in autophagy regulation highlights the tight crosstalk between the two major cellular degradation machineries.

Dysregulation of ubiquitin-mediated autophagy process has been implicated in many disease states, such as neurodegeneration, infectious diseases, liver diseases and metabolic syndromes. With the important role of autophagy in maintaining normal physiology and homeostasis, it is expected to uncover further linkages between dysregulation of ubiquitin-mediated autophagy pathways and various human diseases, especially for age-related diseases. In this regard, targeting of these pathways by modulating the activity of E3 ligase or DUB could be exploited as a strategy for disease intervention and has been an area receiving considerable attention. For example, the small molecular inhibitor of USP10 and USP13, called spautin-1, is capable of antagonizing the ubiquitination and degradation of Beclin-1 and p53, two tumor suppressor proteins, and therefore is a promising anti-cancer agent [37]. In the future, an improved understanding of how ubiquitin-mediated autophagy regulation contributes to the pathology of human diseases and the development of less toxic and more specific agents will benefit more patients.

## Data Availability

Not applicable

## Abbreviations

ATG: Autophagy-related
DUB: Deubiquitinating enzymes
LIR: LC3-interacting region
PD: Parkinson’s disease
PE: Phosphatidylethanolamine
SCA: Spinocerebellar ataxia
TLR: Toll-like receptor
UBA: Ubiquitin binding domain
UPS: Ubiquitin-proteasome system
